# Smoking habit as a risk amplifier in chronic kidney disease patients

**DOI:** 10.1038/s41598-021-94270-w

**Published:** 2021-07-20

**Authors:** Michele Provenzano, Raffaele Serra, Ashour Michael, Davide Bolignano, Giuseppe Coppolino, Nicola Ielapi, Giuseppe Filiberto Serraino, Pasquale Mastroroberto, Francesco Locatelli, Luca De Nicola, Michele Andreucci

**Affiliations:** 1 Renal Unit, Department of Health Sciences, “Magna Graecia” University of Catanzaro, Viale Europa – Campus “Salvatore Venuta”, 88100 Catanzaro, Italy; 2 Interuniversity Center of Phlebolymphology (CIFL), “Magna Graecia” University of Catanzaro, Catanzaro, Italy; 3 Department of Medical and Surgical Sciences, “Magna Graecia” University of Catanzaro, Catanzaro, Italy; 4 Department of Public Health and Infectious Disease, “Sapienza” University of Rome, Rome, Italy; 5 Department of Experimental and Clinical Medicine, “Magna Graecia” University of Catanzaro, Catanzaro, Italy; 6 Nephrology Department, Alessandro Manzoni Hospital, Past Director, Lecco, Italy; 7 Renal Unit, University of Campania “Luigi Vanvitelli”, Naples, Italy

**Keywords:** Medical research, Nephrology, Risk factors

## Abstract

Several studies showed the association between non-traditional risk factors [proteinuria and estimated Glomerular Filtration Rate (eGFR)] and cardiovascular (CV) and renal outcomes. Nevertheless, the etiologic role of traditional CV risk factors in referred CKD patients is less defined. Herein, we examined the association between smoking habit and CV events, mortality and CKD progression. We undertook an observational analysis of 1306 stage III–V CKD patients. Smoking habit was modeled as a categorical (never, current or former smokers) and continuous (number of cigarettes/day) variable. Mean eGFR was 35.8 ± 12.5 mL/min/1.73 m^2^. Never, current and former smokers were 61.1%, 10.8% and 28.1%. During a median follow-up of 2.87 years, current and former smokers were at significant risk for CV events (HRs of 1.93 [95% CI, 1.18–3.16] and 1.44 [95% CI, 1.01–2.05]) versus never smokers. Current smokers were at increased mortality risk (HR 2.13 [95% CI, 1.10–4.11]). Interactions were found between former smokers and proteinuria (*p* = 0.007) and diabetes (*p* = 0.041) for renal risk, and between current smokers and male gender (*p* = 0.044) and CKD stage V (*p* = 0.039) for renal and mortality risk. In referred CKD patients, smoking habit is independently associated with CV events and mortality. It acts as a risk “amplifier” for the association between other risk factors and renal outcomes.

## Introduction

Optimizing risk stratification of chronic kidney disease (CKD) patients referred to tertiary nephrology care remains an important challenge for contemporary Nephrology research, because the identification of high-risk subjects would allow nephrologists to detect those who require a stricter monitoring in terms of number of visits as well as to improve outcomes with timely planning of conservative treatment or eventual renal substitutive therapy^1,2^. Several risk prediction models have been developed for CKD patients, with the aim of improving the risk prediction of cardiovascular (CV) and renal events^3^. More importantly, a great effort has been made to establish the additional role of kidney parameters, namely eGFR and proteinuria, on top of the traditional risk factors. The cutting-edge work of the CKD-PROGNOSIS Consortium represents an example of this effort^4,5^. It has been well demonstrated that eGFR and proteinuria are strong predictors of worse outcome in CKD patients independently from each other and from traditional risk factors, this association being true for CV events, all-cause mortality and renal events^1–5^. On the other hand, despite the fact that traditional risk factors, such as lipid, hypertension, smoking habit have been shown to be associated with increased CV risk in the general population, what their exact role is in addition to kidney measures in patients already diagnosed with CKD needs to be better clarified. With respect to smoking habit, several studies in humans have shown that cigarette smoking exerts renal and systemic toxic effects by worsening endothelial dysfunction, oxidative stress, and glomerulosclerosis^6–8^. Moreover, the combination of cigarette smoking and CKD could represent an extremely high-risk state since the use of tobacco impairs hemodynamic parameters, such as blood pressure and peripheral vascular resistance, and is associated with the development of albuminuria which in turn represents another biomarker of CV disease^8–10^. This notwithstanding, previous studies have considerably explored the association of smoking habit with CKD progression while data on the etiologic effect of smoking on CV risk and mortality in the CKD setting are limited^8,11–13^. Therefore, the aims of this observational, prospective study were to assess the following endpoints in a large cohort of patients referred to nephrology clinics: (1) the association of smoking habit with future fatal and non-fatal CV events, all-cause mortality and renal events; (2) the association between number of cigarettes/day and the aforementioned endpoints.

## Results

We studied 1306 patients with a complete CV risk profile (Table 1).

**Table 1:** Basal characteristics of patients overall and by smoking habit (never, former, current).

	Overall (n = 1.306)	Smoking habit			
		Never (n = 798)	Former (n = 367)	Current (n = 141)	*p*
Age, years	67.6 ± 11.8	68.0 ± 12.3	68.5 ± 10.0	63.3 ± 12.4	< 0.001
Male gender, %	65.2	51.3	89.7	80.1	< 0.001
Diabetes, %	24.6	23.6	27.8	22.0	0.223
CV disease, %	27.2	20.2	43.3	24.8	< 0.001
Body mass index, kg/m^2^	27.3 ± 4.5	27.3 ± 4.7	27.5 ± 4.2	26.2 ± 3.9	0.018
**Primary kidney disease, %**					0.003
HTN	32.4	29.2	37.9	36.2	
DN	16.4	16.4	16.7	14.9	
GN	9.3	8.9	8.2	14.2	
TIN	6.1	5.3	8.5	6.8	
PKD	2.6	3.3	2.1	1.4	
Other/unknown	33.3	37.0	24.1	28.9	
Blood pressure, mmHg	140 ± 18/80 ± 9	140 ± 18/80 ± 9	140 ± 19/80 ± 10	139 ± 19/81 ± 11	0.874/0.230
eGFR, mL/min/1.73 m^2^	35.8 ± 12.5	34.8 ± 12.5	37.3 ± 12.0	37.6 ± 12.8	0.001
Calcium, mg/dL	9.3 ± 0.7	9.3 ± 0.7	9.3 ± 0.6	9.2 ± 0.6	0.399
Phosphorus, mg/dL	3.6 ± 0.8	3.7 ± 0.8	3.5 ± 0.7	3.7 ± 0.7	0.003
PTH (pg/ml)	92 [55–156]	106 [64–179]	81 [50–120]	83 [48–158]	0.002
Serum Albumin, g/dL	4.0 ± 0.6	4.0 ± 0.6	3.9 ± 0.5	4.0 ± 0.5	0.710
Hemoglobin, g/dL	12.8 ± 1.8	12.5 ± 1.8	13.1 ± 1.7	13.3 ± 2.1	< 0.001
Cholesterol, mg/dL	201 ± 48	203 ± 49	197 ± 46	201 ± 50	0.096
LDL-cholesterol, mg/dL	119 ± 40	122 ± 41	115 ± 38	117 ± 41	0.015
Uprot, g/24 h	0.20 [0–0.99]	0.20 [0–0.70]	0.20 [0–1.00]	0.30 [0–1.40]	0.068
Antihypertensives, number	2.15 ± 1.26	2.08 ± 1.28	2.34 ± 1.20	2.08 ± 1.28	0.007
RAASi, % pts	57.2	54.5	61.0	62.4	0.047
Statins, %pts	36.9	33.5	44.7	35.1	0.003

*CV* cardiovascular, *HTN* hypertensive nephropathy, *DN* diabetic nephropathy, *GN* glomerulonephritis, *TIN* tubulointerstitial nephropathies, *PKD* polycystic kidney disease, *PTH* parathyroid hormone, *LDL* low-density lipoprotein, *RAASi* renin–angiotensin–aldosterone-system inhibitors.

Patients were recruited from 79 nephrology centers with a median of 20 patients/center [IQR: 13–27 patients]. As expected, the whole cohort was characterized by a high-risk profile (being patients referred to tertiary nephrology care) as testified by the consistent frequency of diabetes (24.6%), background CV disease (27.2%) as well as by the high Body Mass Index and blood pressure levels on average. In the entire population we registered 798 (61.1%) never smokers, 141 (10.8%) current smokers and 367 (28.1%) former smokers. Current smokers were significantly younger than former and never smokers (63.3 years-old vs. 68.5 and 68.0 years-old, respectively; *p* < 0.001) and were more frequently diagnosed with glomerulonephritis (GN) compared with the other two categories (14.2% of GN patients who were current smokers vs. 8.2% and 8.9% who were former and never smokers respectively, *p* = 0.003). Current and former smokers were overall more frequently males, with significantly higher eGFR and hemoglobin and treated more frequently with renin–angiotensin–aldosterone inhibitors compared with never smokers (Table 1). Median proteinuria levels were slightly higher, albeit not significantly, in current smokers (median of 0.3 [IQR 0–1.4] g/24 h) than former (median 0.2 [IQR 0–1.0] g/24 h) and never smokers (median 0.2 [IQR 0–0.7] g/24 h). Previous CV disease (including acute myocardial infarction, stroke, chronic heart failure, peripheral vascular disease) was more prevalent in the former smokers category than in the current or never smokers categories (43% vs. 20–25%, *p* < 0.001). LDL-cholesterol was significantly lower in current (117 ± 41 mg/dL) and former smokers (115 ± 38 mg/dL) versus never smokers (122 ± 41 mg/dL) with *p* = 0.015, in agreement with a wider use of statins in these two categories.

### Follow-up data

At survival analysis, follow-up lasted 2.87 [IQR 1.57–3.03] years. During the study follow-up, we registered 178 major CV events (164 non-fatal events and 57 CV deaths with 43 deaths occurring after a non-fatal CV event), 95 all-cause deaths and 181 renal events (52 chronic renal replacements and 125 eGFR halving). The detailed distribution of non-fatal CV events is reported in Table 2.

**Table 2:** Distribution of non-fatal cardiovascular events which occurred during study follow-up.

Event type	Frequency N (%)
Myocardial infarction	43 (24.2)
Stroke	14 (7.9)
Heart failure	48 (27.0)
Coronary revascularization	26 (14.6)
Peripheral arterial vascular disease	33 (18.5)

**Figure 1:**
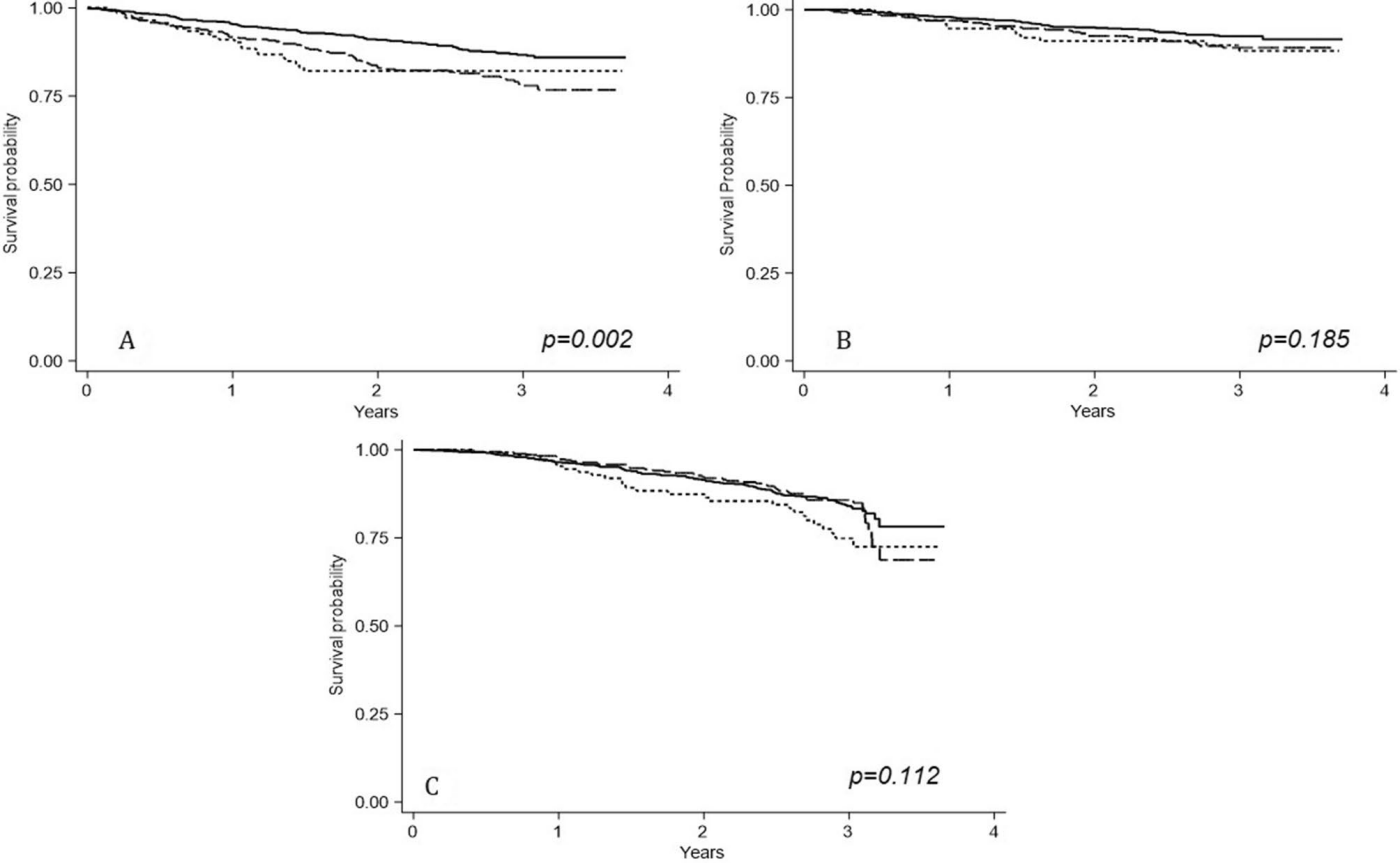
Survival probabilities of CV fatal/non-fatal events (**A**), all-cause death (**B**) and Renal events (**C**) according to smoking habit categories. Continuous line refers to never smokers; long-dashed line refers to former smokers; short-dashed line refers to current smokers.

The crude incidence rate for CV fatal and non-fatal events was significantly higher in former and current smokers, being 8.17 (95% CI 6.34–10.38) and 7.38 (95% CI 6.34–10.38) per 100 pts-y, respectively. Crude rates of all-cause death and renal events did not differ between smoking habit categories despite a tendency of current smokers to have an apparent higher rate for renal events compared with never and former smokers (Fig. 1).

At adjusted Cox analyses, which accounted for other cardiorenal risk factors including age, gender, proteinuria and eGFR, current and former smokers were at significant increased risk, by 93% and 44% respectively, for fatal and non-fatal CV events (Table 3).

**Table 3:** Relative risks for cardiovascular fatal and non-fatal events, all-cause death and renal events.

	CV fatal and non-fatal events			All-cause death			ESKD		
	HR	95% CI	*p*	HR	95% CI	*p*	HR	95% CI	*p*
Age, years	**1.04**	**1.02–1.06**	**< 0.001**	**1.07**	**1.04–1.09**	**< 0.001**	**0.97**	**0.96–0.98**	**< 0.001**
Gender, male versus female	1.40	0.97–2.04	0.074	1.06	0.65–1.74	0.818	1.31	0.95–1.83	0.104
eGFR, mL/min/1.73m^2^	**0.98**	**0.96–0.99**	**< 0.001**	**0.98**	**0.96–0.99**	**0.009**	**0.92**	**0.91–0.94**	**< 0.001**
Proteinuria, g/24 h	1.04	0.96–1.12	0.380	1.08	0.99–1.18	0.090	**1.16**	**1.11–1.20**	**< 0.001**
**Smoking habit, never**	Ref	Ref	Ref	Ref	Ref	Ref	Ref	Ref	Ref
Current smokers	**1.93**	**1.18–3.16**	**0.009**	**2.13**	**1.10–4.11**	**0.024**	1.30	0.82–2.06	0.268
Former smokers	**1.44**	**1.01–2.05**	**0.042**	1.30	0.79–2.15	0.295	0.98	0.68–1.42	0.927
Previous CV disease, yes versus no	**2.03**	**1.48–2.79**	** < 0.001**	**2.28**	**1.48–3.51**	** < 0.001**	**1.51**	**1.05–2.16**	**0.024**
LDL-Cholesterol, mg/dL	1.01	0.99–1.01	0.071	1.00	0.99–1.01	0.977	1.01	0.99–1.01	0.900
Systolic blood pressure, mmHg	0.99	0.99–1.01	0.500	0.99	0.99–1.01	0.554	**1.01**	**1.00–1.02**	**0.010**
Diabetes, yes versus no	**1.62**	**1.18–2.23**	**< 0.001**	0.99	0.62–1.58	0.968	1.30	0.92–1.82	0.136

*HR* hazard ratio, *CV* cardiovascular, *ESKD* end-stage-kidney-disease, *Ref* reference category. Estimates with p value <0.05 are shown in bold.

Current smokers were also at twofold higher risk for all-cause death compared with never smokers (HR 2.13; 95% CI 1.10–4.11, *p* = 0.024), whereas former smokers were apparently not at increased risk versus never smokers (HR 1.30; 95% CI 0.79–2.15; *p* = 0.295). With respect to the renal outcome, smoking habit did not show an independent association with the composite endpoint of ESKD/50% eGFR decline (Table 3). Overall, the model with smoking habit outperformed the model without smoking habit for the prediction of CV events (LR test *p* = 0.018; difference between *c*-index *p* = 0.001). A weaker but still significant improvement in model discrimination was also shown for the mortality endpoint (Table 4).

**Table 4:** Models performance and additional predictive value of smoking habit, when added to the fully adjusted Cox model, on the study endpoints.

	CV fatal and non-fatal events			All-cause death			ESKD		
	Model 1	Model 2	*p*	Model 1	Model 2	*p*	Model 1	Model 2	*p*
LR test	–		**0.018**	–		0.095	–		0.507
c-index	**0.705 (0.669–0.741)**	**0.716 (0.678–0.750)**	**0.001**	**0.684 (0.644–0.722)**	**0.692 (0.654–0.731)**	**0.002**	0.672 (0.631–0.714)	0.673(0.630–0.714)	0.943
R^**2**^	**66.0 (59.8–78.0)**	**69.9 (64.1–82.1)**	**–**	**72.3 (62.8–89.3)**	**74.2 (65.6–90.0)**	**–**	**63.2 (50.1–84.6)**	**67.1 (52.3–87.1)**	**–**
AIC	**2373**	**2330**	**–**	**2227**	**2204**	**–**	**2375**	**2334**	**–**
BIC	**2425**	**2393**	**–**	**2279**	**2266**	**–**	**2421**	**2386**	**–**

LR test (Likelihood Ratio test). Comparisons are made between Model 1 (without smoking habit) and Model 2 (including smoking habit). Model 1 and 2 were adjusted for all the covariates included in Table 3.

*AIC* akaike information criterion, *BIC* Bayesian information criterion, *R*^*2*^ Royston’s modification of Nagelkerke’s R^2^. Estimates with p value <0.05 are shown in bold.

**Figure 2:**
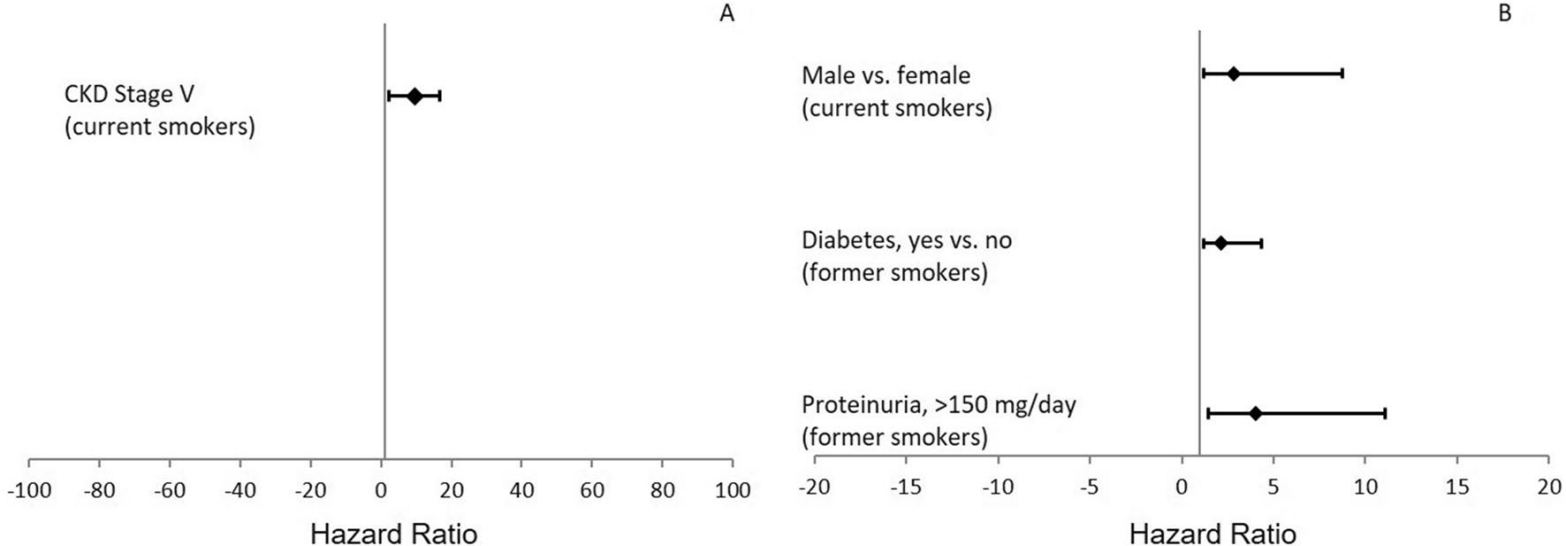
Interaction effects between smoking habit and other clinical/demographic variables for the all-cause death (**A**) and renal (**B**) endpoints.

As shown in Table 4, the explained variation (R^2^) of the models increased with the addition of smoking habit to the other variables. Conversely, AIC and BIC decreased after inclusion of smoking habit, thus demonstrating a better overall performance of the model with smoking. When interaction effects were tested, we found a positive effect modification between smoking habit and proteinuria, gender and diabetes on the risk of ESKD and a negative interaction between smoking habit and eGFR. After stratifying eGFR levels according to CKD stages, we detected a significant positive interaction between current smokers and CKD Stage V (*β* = 2.27, *p* = 0.039) for the risk of all-cause death, between former smokers and proteinuria > 150 mg/day (*β* = 1.39, *p* = 0.007) and the presence of type II diabetes (*β* = 0.75, *p* = 0.041) for the risk of ESKD/50% eGFR decline. Moreover, being a current smoker modified the association between gender and renal outcome with a higher risk in male versus female patients (*β* = 1.03, *p* = 0.044). Hazard Ratios of the effect modifications are depicted in Fig. 2.

For sensitivity analysis, we built a model with smoking habit modeled as a binary variable (current smokers versus never or former smokers) and results remained unchanged. Current smokers were indeed at increased risk for CV fatal/non-fatal events (HR: 1.63, 95% CI 1.03–2.59, *p* = 0.037) and all-cause death (HR: 1.89, 95% CI 1.02–3.49, *p* = 0.042) but not with renal endpoint (HR: 1.31, 95% CI 0.84–2.02, *p* = 0.233). A further sensitivity analysis was performed by adding to the full model, presented in Table 3, the years of smoking for both current and former smokers. According to this analysis, the risk for CV fatal and non-fatal events and all-cause death increased by 2% (*p* = 0.013) and 3% (*p* = 0.027), respectively, for each year of smoking. Conversely, smoking duration did not influence the renal risk (*p* = 0.706). When the effect of cigarette number/per day on cardiorenal risk was evaluated, CV and mortality risk increased by 4% for each cigarette per day consumed in current smokers, whereas CV events risk only was increased by 1% for one more cigarette, in former smokers. Number of cigarettes was not associated with an increased risk for renal events in any case (Table 5).

**Table 5:** Cardiovascular, mortality and renal risk by number of cigarettes consumed per day.

	CV fatal and non-fatal events			All-cause death			ESKD		
	HR	95% CI	*p*	HR	95% CI	*p*	HR	95% CI	*p*
**Current smokers**									
Cigarettes, number/day	**1.04**	**1.01–1.06**	**0.001**	**1.04**	**1.02–1.07**	**0.001**	1.02	0.99–1.04	0.173
**Former smokers**									
Cigarettes, number/day	**1.01**	**1.00–1.02**	**0.036**	1.00	0.99–1.02	0.442	1.01	0.99–1.01	0.643

Models (with current or former smokers, separately) are adjusted for all the covariates included in Table 3.

*HR* hazard ratio, *CV* cardiovascular, *ESKD* end-stage-kidney-disease. Number of cigarettes variable was log-transformed due to the skewed distribution. Estimates with p value <0.05 are shown in bold.

Measures of model performance for models included in Table 5 are reported in Supplementary Table S2. Tests for proportional hazard assumptions were not significant for all variable and for all the endpoints (Table 6, Supplementary Table S3 and Supplementary Figures S1–S4).

**Table 6:** Tests of proportional hazard assumptions in survival models (Derived from Table 3).

Variables	CV fatal and non-fatal events	All-cause death	ESKD
	*p*	*p*	*p*
Age	0.567	0.160	0.122
Gender	0.176	0.614	0.148
eGFR	0.277	0.256	0.195
Proteinuria	0.192	0.254	0.663
Current smokers	0.800	0.664	0.728
Former smokers	0.475	0.990	0.189
Previous CV disease	0.115	0.258	0.436
LDL-cholesterol	0.306	0.600	0.361
Systolic blood pressure	0.804	0.231	0.597
Diabetes	0.821	0.578	0.740
Global test	0.445	0.401	0.318

**Figure 3:**
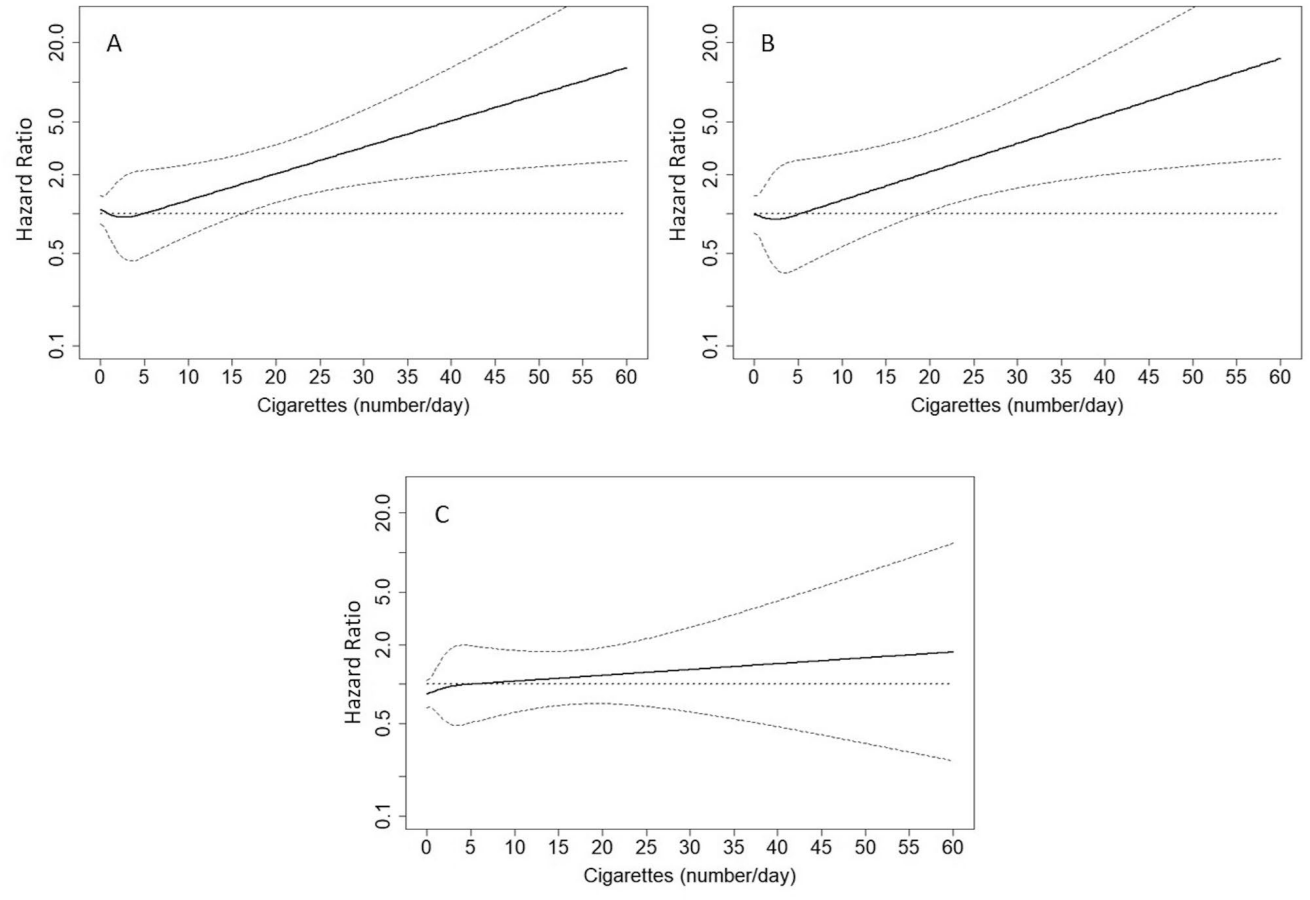
Hazard ratios (solid thick lines) and 95% confidence intervals (dashed thin lines) for cardiovascular fatal and non-fatal events (**A**), all-cause death (**B**) and renal events (**C**). Hazard ratios (HRs) were modeled by means of restricted cubic spline (RCS) due to the nonlinear association between number of cigarettes and the endpoints. HRs refers to current smokers and were adjusted for all the covariates included in Table 3.

Thus, there was no compelling evidence of departures from the proportionality assumption. Variance inflation factors for the variables included in Tables 3 and 5 were all below 5, thus excluding issues of multicollinearity (Supplementary Table S4). Restricted cubic spline analyses showed that the risk for CV fatal and non-fatal events became significant early at 15 cigarettes/day whereas risk for all-cause death started at 20 cigarettes/day in current smokers (Fig. 3A, B). We did not find a significant threshold for the number of cigarettes in association with renal events (Fig. 3C).

## Discussion

Chronic Kidney Disease represents a major global public-health problem, affecting almost 700,000 individuals worldwide^14^. Moreover, the Global Burden of Kidney Disease reported a doubling in incidence and prevalence of CKD which increased by 89% and 87%, respectively, over the past 3 decades^15–17^. This trend has been attributed to some extent to the concomitant population ageing and the consequent increase in incidence of comorbidities^16,18^. Accordingly, the mortality rate due to CKD has increased by about 4.4% in the same time interval^15^. All these evidences are alarming if we consider that the overall CV mortality rate has declined on the global scale as result of the multiple prevention strategies applied to the general population including a better control of blood pressure levels, improved physical activity and a wider use of statins^19,20^. Hence, a further effort is required to reduce the social burden as well as to improve the prognosis of CKD patients. Referred CKD patients constitute a peculiar population with a shorter life expectancy driven by the high risk of renal function decline, overall mortality and CV risk^21–24^. Prevalence of CV disease in CKD population ranged from 30 to 50% of patients, and the CV events rate was even higher than the mortality rate^25–31^. Despite a number of clinical trials that have been conducted so far to optimize therapeutical control of CV risk in CKD^31–34^, similar attention should be directed toward better control of behavioral, demographic and other modifiable clinical risk factors as well as to the implementation of novel biomarkers^35–38^. In our observational analysis we found that smoking habit is independently associated with a significant increased risk for CV fatal and non-fatal events and mortality in a large cohort of already diagnosed CKD patients referred to Nephrologists. Furthermore, despite the finding that variable smoking did not predict renal progression independently, smoking habit acts as an “amplifier” of the association between other covariates and renal outcome. This is testified by the effect of modification of smoking habit on 24 h-proteinuria, diabetes and male gender for renal risk. Overall, all this information is novel since the previous large prospective studies are limited, derived from small samples or, even more importantly, have been carried out in populations at increased risk for CKD. High-risk populations differ, for basal characteristics and prognosis, from patients referred to nephrologists^1,39^. In a cohort of 147 CKD individuals followed in the renal outpatients clinic, Jungers et al. showed that cigarette smoking was more common in patients who developed CV events during follow-up as compared with those who did not^40^. However, the authors did not provide a comprehensive risk profile and prognostic estimates of smokers versus non-smokers among the CKD patients and the small sample size did not allow to observe a sufficient and heterogeneous number of CV events because the analysis was limited to myocardial infarction and stroke. Of note, a more extended analysis from the Cardiovascular Health Study, which included nearly 6.000 subjects, showed that current smoking predicted CV deaths in a subgroup of patients with CKD whereas it was not significantly associated with the outcome in patients without CKD. These observations are of interest; however, they cannot be compared to our results. In fact, patients enrolled in this previous study were selected from the general population and were older than 65 years, which makes these data not generalizable to the whole CKD population^41^. In our cohort, 32.9% patients were < 65 years of age and suffered from a wide spectrum of renal diagnoses (including glomerulonephritis, polycystic kidney disease, diabetic nephropathy), that represent, considering previously published literature, an important and peculiar feature of CKD patients followed by Nephrologists^42^. In the Atherosclerosis Risk in Communities (ARIC) study current smoking status increased CHD risk by 65 up to 91%^11^. However, in the ARIC cohort only 391 out of 14,856 patients (2.6%) had CKD and thus the overall cohort showed a different basal risk profile compared with our patients. In fact, about 48.5% of patients included in the ARIC study had eGFR > 90 mL/min/1.73 m^2^, whereas proteinuria was not included in the prediction model^11^. In the Chronic Renal Insufficiency Cohort (CRIC) study, a large cohort of patients with mild-moderate CKD, tobacco smoking displayed a higher risk for both mortality and CKD progression^12^. Interestingly, we have expanded the previous observations by assessing the association between smoking habit and CV events, mortality and renal outcomes in CKD patients. Moreover, all these previous studies examined the relationship between smoking and CV or renal events, but there are no studies that present the interaction effect of smoking with other risk factors. An attempt to stratify the prognostic effect of smoking by diabetes or previous CV disease was performed on the SHARP (Study of Heart and Renal Protection) trial. However, the risk stratification was not reported for the renal outcome, and in fact smoking seemed to play a protective effect on ESKD in this study, likely because of a collider bias problem which occurred when smoking habit was adjusted for other confounders in that analysis^43^. We have also described for the first time a dose–effect relationship between the number of cigarettes consumed and the occurrence of worse outcomes. Therefore, smoking habit plays a negative role in determining the future of CKD patients. The detrimental effects of smoking on kidney biology and function may rely on several plausible mechanisms, including endothelial dysfunction, oxidative stress, impaired lipid metabolism and activation of fibrotic mediators such as TGF-β, which, if considered together, increase individual risk^44–48^. In addition, cigarette smoke contains a vast amount of toxins which promote pathological changes in renal vasculature^2,7,49^. The pathophysiological mechanisms of smoking-associated cardiorenal damage should be combined with the public health dimension of the problem. In the period between 1980 and 2012, the global age-standardized prevalence of tobacco smoking was decreased by 25% and 42% for men and women, respectively. Nevertheless, such a declining trend was biphasic with a first period (1996–2006) of sharp decline followed by a period (2006–2012) of slower reduction. Simultaneously, the tobacco market increased, with a 26% increase in the total number of cigarettes consumed^50^. These data converge toward the need of making a further effort to reduce as much as possible the prevalence of patients still smoking within the population. This is particularly true if considering that numerous developed countries, including in Eastern Europe, Italy, Greece, Ireland, are considered to be both at high prevalence and high consumers of cigarettes (≥ 20/day)^50^. The presence of CKD even worsens the risk for CV incidents in our study as it starts when consuming less than 20 cigarettes/day. The current study has some strengths and limitations. As for the strengths, we present a comprehensive study that evaluates predictive ability of smoking habit in a large cohort of referred CKD patients and evaluated, for the first time, the effect of modifications in smoking in combination with other risk factors. On the other hand, although this is a multicenter study, our analysis was limited to the Italian population, and the results may be difficult to generalize to other multiethnic populations. Furthermore, the exposure-outcome association could have been influenced by a number of unknown confounders, so that a residual confounding may still be present. In conclusion, smoking habit has an independent negative effect on CV and mortality outcome of CKD patients, even after accounting for other major traditional and non-traditional CV risk factors such as proteinuria, eGFR, diabetes and previous CV disease. Former and current smoking have an indirect effect by strengthening the association of diabetes, proteinuria and male gender on renal endpoints and CKD stage V with mortality. A small number of cigarettes consumed per day, even less than 1 pack, are sufficient to increase CV and mortality risk. Given this scenario, more studies are needed to understand how to manage CKD patients with previous exposure to smoking as they may present a significant residual CV risk.

## Methods

### Study design and procedures

The present study has been carried-out on the SIR-SIN cohort (“Italian study on multiple predictors of outcome epidemiology of chronic renal insufficiency in Italy”) conceived by the Italian Society of Nephrology (SIN). The SIR-SIN is an observational, multicenter, prospective study started in June 2004 with the aim of recruiting data from the whole national area about demographic and clinical features, treatment, and prognosis of newly diagnosed CKD patients on conservative treatment. The last patient was enrolled in October 2009. Patients were followed for 3 years or until reaching 200 patients with doubling of serum creatinine per Center. Details of the study protocol have been reported elsewhere^51^. In short, patients were included in the study if they were diagnosed with CKD stage III–V according to K/DOQI criteria (i.e. eGFR < 60 mL/min/m^2^ for at least 3 months), had a life expectancy > 6 months and were over 18 years old. The main exclusion criteria were presence of acute kidney injury, patients who were already on renal replacement therapies (dialysis or kidney transplant) and active malignancy. Patients with no available lipid profile were also excluded. Data were collected from 92 Nephrology centers, out of 100 that previously had accepted to be included in the project. The study received approval from local ethical committees and was performed in accordance with the Good Clinical Practice guidelines. The final experimental protocol was validated and approved by the Ethical Committee of Calabria Region–Area Center Section [Protocol ID: DB 02 03 (INN26I2002)]. All methods were performed in accordance with the relevant guidelines and regulations. At the referral visit, all patients gave informed consent and participating nephrologists collected information regarding demographic characteristics, medical history including cardiovascular risk factors, any prescribed therapy, clinical and laboratory data, and registered all the information into electronic case report forms. Cigarette smoking habit was determined through the use of standardized questionnaires. Patients were labeled as “current” smokers if they responded affirmatively to the question “Have you smoked cigarettes in the last week?”. Former smokers were so-defined if they smoked at least 100 cigarettes in their lifetime. Estimated GFR was computed with the CKD-EPI formula by reducing serum creatinine levels by 5% according to Skali et al.^52^, because they were not standardized to isotope-dilution mass spectrometry values. After referral visit, patients were followed for the primary endpoints which were major CV events (CV death or non-fatal CV events requiring hospitalization among acute myocardial infarction, stroke, heart failure, coronary revascularization, peripheral arterial vascular disease), all-cause death and renal events. Renal events were End-Stage-Kidney-Disease (ESKD), namely the need for chronic dialysis, pre-emptive kidney transplant, or 50% eGFR decline.

### Statistical analysis

Continuous variables are reported as either mean ± standard deviation (SD) or median and interquartile (IQR) range based on their distribution. Comparison among smoking habit categories was assessed by ANOVA or the Kruskal–Wallis test depending on the distribution. Categorical variables are reported as percentages and comparisons between categories were assessed by Chi-square test. In our original cohort, the missing data were as follows: serum calcium (n = 64), parathyroid hormones (n = 85) and serum albumin (n = 84). We first tested the differences between patients with one or more missing variables and those with complete data with reference to demographics, clinical characteristics and outcomes. As we did not find any differences, we imputed the missing data to include all patients in all analyses, including survival models by implementing multiple imputation^53^. For survival analysis, follow-up was started from the referral visit and its median value was estimated by the inverse Kaplan–Meier approach. Patients lost to follow up were right censored at the time of the last outpatient visit. We computed incidence rates of CV fatal and non-fatal events, all-cause mortality and ESKD. Rates were reported as number of events/person-time and 95% confidence intervals (CIs) were calculated assuming a Poisson distribution. Crude survival probabilities according to smoking categories were computed by the Kaplan–Meier approach and the log-rank test was calculated. Cox-proportional-hazard models were run to compute the hazard ratio (HR) and 95% CI because the cause-specific relative hazards are more appropriate for studying the cause of diseases in the case of competing events^54^. In fact, in our cohort, ESKD, all-cause death before ESKD and CV fatal and non-fatal events are competitive endpoints, lasting the follow-up until each event occurs. Models were adjusted for the same basal covariates (age, gender, eGFR, proteinuria, history of CV disease, LDL-cholesterol, systolic blood pressure and diabetes) that were identified a priori as cardiorenal risk factors based on previous literature^51,54^. To evaluate the predictive utility of adding smoking habit to the full adjusted model we calculated the likelihood ratio test and the difference in discrimination (*c*-index) between a model without and a model with smoking habit. Comparison between *c*-indexes has been computed with 1000 bootstrap replicates and the percentile method^54^. Several metrics of models’ goodness-of-fit have been provided. We computed Akaike information criterion (AIC), Bayesian information criterion (BIC) and R^2^. The R^2^, which quantifies the explained variation of the whole survival model, was estimated by means of Royston's modification of Nagelkerke’s R^2^^55^. Next, we computed the first-order interactions between smoking habit and all the covariates included in the fully adjusted models. To better investigate the meaning of significant interaction effects, several variables have been categorized and interactions between categorical variables and smoking habit have been shown. Continuous eGFR was replaced by a CKD Stage (I–III, IV and V), and continuous 24 h proteinuria was replaced by proteinuria < or ≥ 150 mg/day according to the KDIGO classification^19^. In the survival analysis, the proportional-hazards assumption has been tested based on Schoenfeld residuals and visual plot. Multicollinearity was tested by means of VIF and values greater than 10 were cause for concern^56^. We assessed the dose–effect of number of cigarettes consumed per day on the three study outcomes. Owing to the skewed distribution of the variable ‘number of cigarettes/day’, this variable has been included in the survival models as log-transformed or restricted cubic spline (RCS) with knots at 2 and 5 cigarettes/day, whereas the reference point was 5 cigarettes/day. Knots have been located according to the median distribution of the number cigarettes/day. RCS analysis was also run with the aim of evaluating the risk threshold above which the risk started to increase. Nonlinear associations between ‘number of cigarettes/day’ and the three endpoints (ESKD, all-cause death and CV fatal and non-fatal events) were also tested and retained in the final model if significant nonlinear effects were found. A two-tailed *p* value < 0.05 was considered significant. Data were analyzed using STATA version 14 (StataCorp. College Station, TX, USA) and rms, hmisc packages of R software 3.3.1 (R Foundation for Statistical Computing, Vienna, Austria).

### Ethical approval

Subjects have given their written informed consent. The study protocol has been approved by the research institute’s committee on human research. This paper is not directly related to animal research.

## Supplementary Information

Supplementary Information.

## Data Availability

The datasets generated during and/or analyzed during the current study are available from the corresponding author on reasonable request.
