# Supplementary material for: Smoking habit as a risk amplifier in chronic kidney disease patients
Source: Sci Rep. 2021 Jul 20;11:14778. doi: 10.1038/s41598-021-94270-w (PMC8292329; doi:10.1038/s41598-021-94270-w)
Supplement: Supplementary file 1 — Supplementary Information. [file 41598_2021_94270_MOESM1_ESM.docx]

**Supplementary Data**

**Supplementary Table S1.** Interaction effects between covariates included in Cox models for cardiovascular fatal and non-fatal events, all-cause death and End-Stage-Kidney-Disease risk prediction.

|  | **All-cause death** | **ESKD** |
| --- | --- | --- |
| **Interaction** | **β, p-value** | **β, p-value** |
| Smoking habit*eGFR | -0.05, 0.020 | - |
| Smoking habit*Proteinuria | - | 0.25, <0.001 |
| Smoking habit*gender | - | 1.03. <0.001 |
| Smoking habit*diabetes | - | 0.75, 0.021 |

**Supplementary Table S2.** Discrimination, goodness-of-fit and model performance of current and former smokers (for the models included in Table 5).

|  | **CV fatal and non-fatal events** | **All-cause death** | **ESKD** |
| --- | --- | --- | --- |
| **Current smokers** |  |  |  |
| c-index | 0.724 (0.691-0.768) | 0.725 (0.688-0.773) | 0.706 (0.665-0.731) |
| R^2^ | 72.1 (66.2-82.3) | 75.3 (69.0-84.0) | 68.5 (59.3-79.9) |
| AIC | 2314 | 2193 | 2328 |
| BIC | 2385 | 2249 | 2374 |
| **Former smokers** |  |  |  |
| c-index | 0.709 (0.691-0.768) | 0.707 (0.661-0.757) | 0.687 (0.646-0.729) |
| R^2^ | 65.3 (62.0-81.4) | 70.1 (64.5-82.1) | 67.3 (52.1-87.2) |
| AIC | 2377 | 2200 | 2331 |
| BIC | 2423 | 2257 | 2386 |

**Supplementary Figure S1** Tests of proportional hazard assumptions for the variables included in Table 3 and cardiovascular fatal and non-fatal events as endpoint.


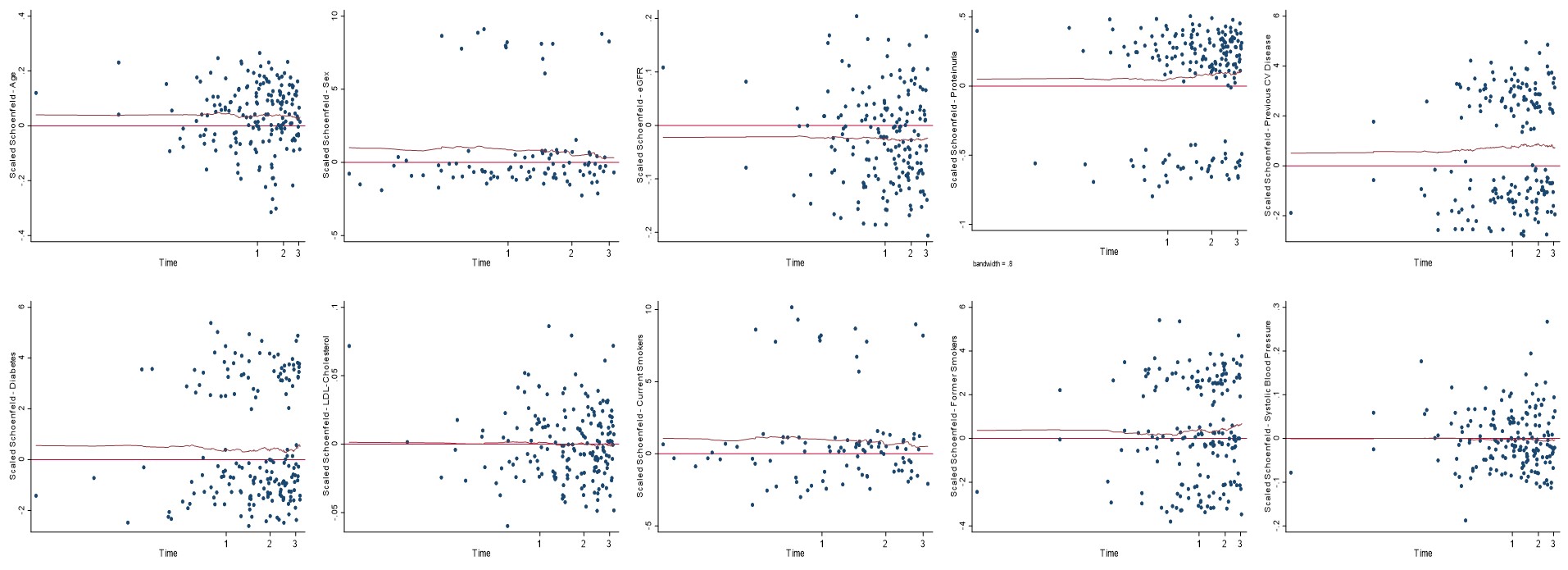


**Supplementary Figure S2** Tests of proportional hazard assumptions for the variables included in Table 3 and all-cause death as endpoint.


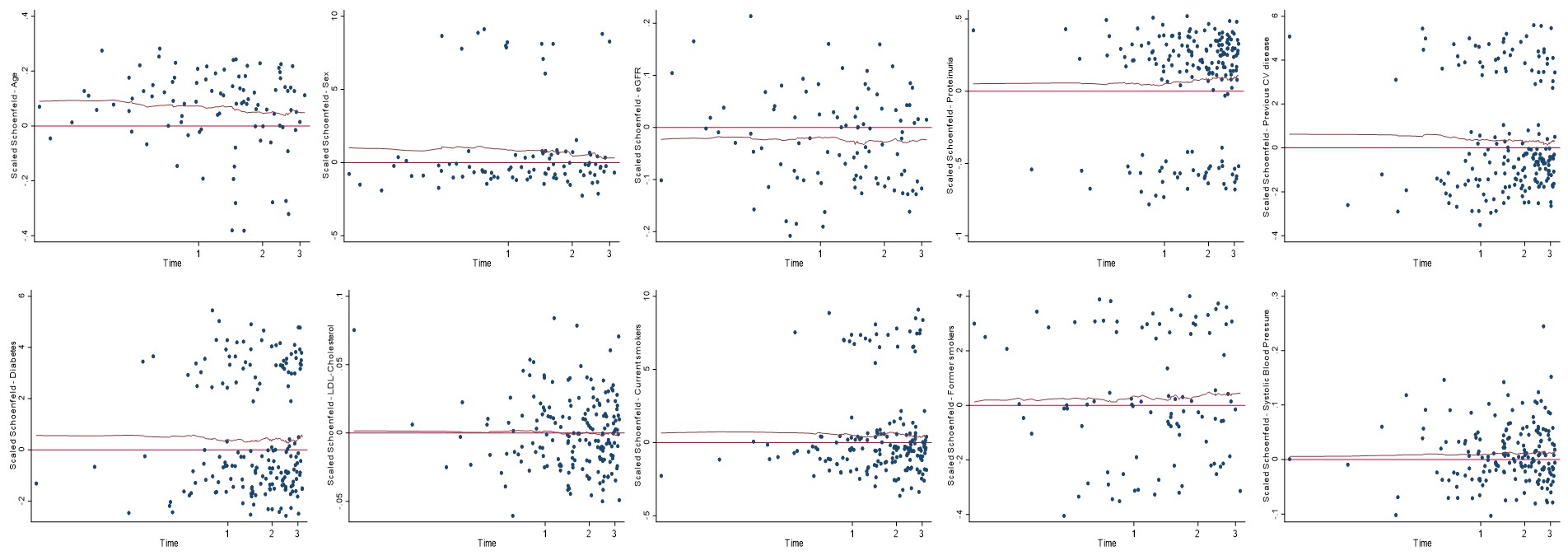


**Supplementary Figure S3** Tests of proportional hazard assumptions for the variables included in Table 3 and End-Stage-Kidney-Disease as endpoint.


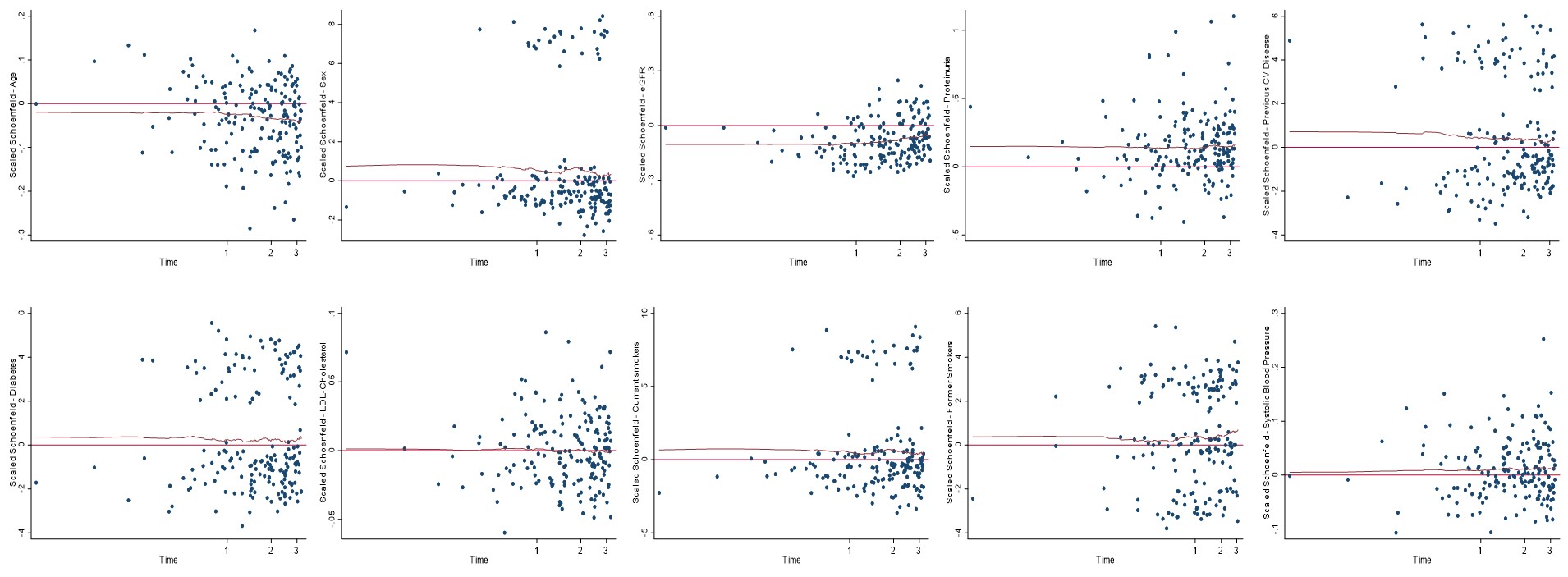


**Supplementary Figure S4** Tests of proportional hazard assumptions for the variable number of cigarettes/day included in Table 5 (current smokers are depicted in panel A and former smokers are depicted in panel B) for all the study endpoints.


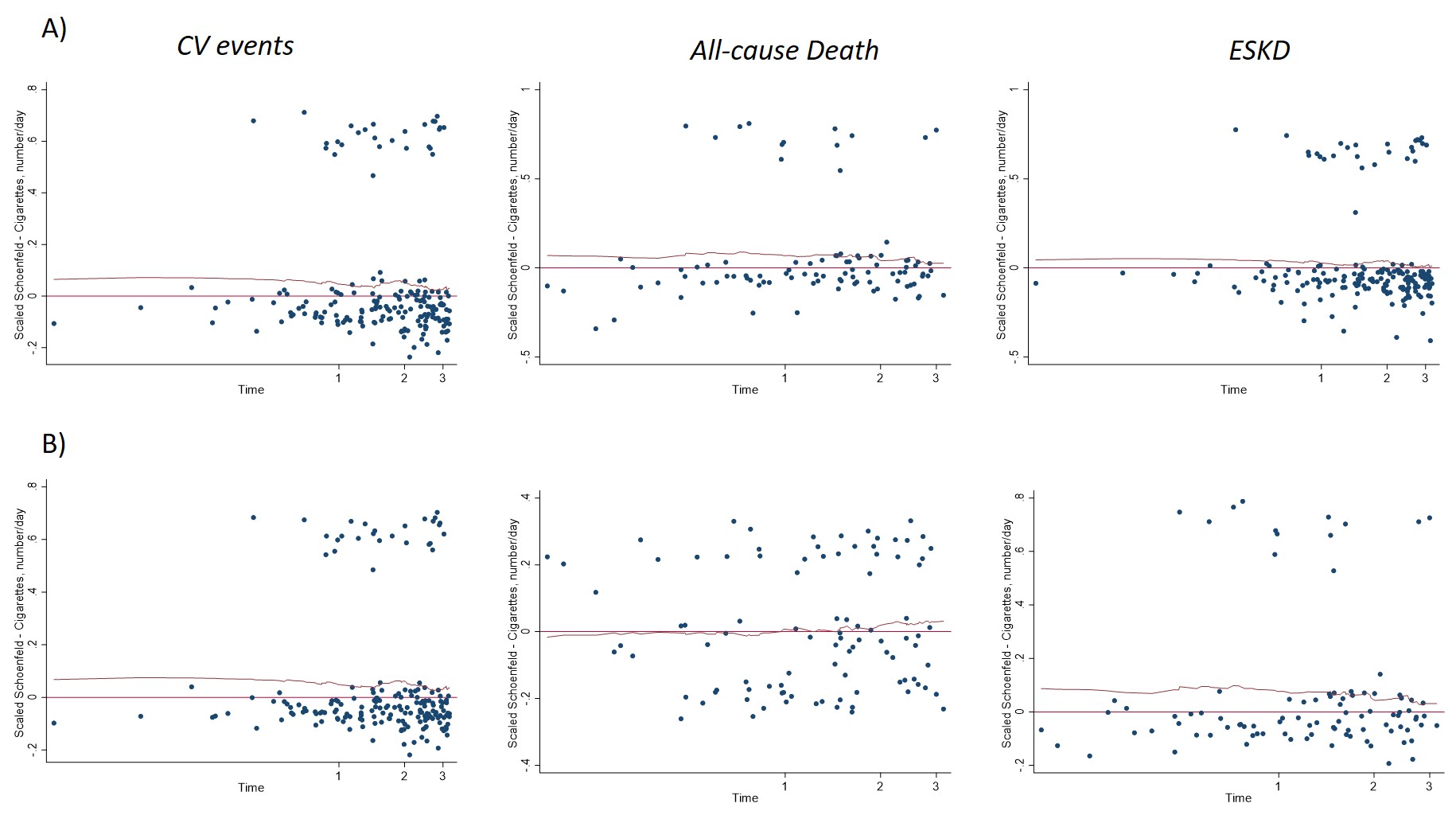


**Supplementary Table S3.** Tests of proportional hazard assumptions in survival models (Applied to models of Table 5).

| **Variables** | **CV fatal and non-fatal events** | **All-cause death** | **ESKD** |
| --- | --- | --- | --- |
|  | ***p*** | ***p*** | ***p*** |
| **Current smokers** |  |  |  |
| Cigarettes, *number/day* | 0.703 | 0.616 | 0.867 |
| Global test | 0.428 | 0.470 | 0.392 |
| **Former smokers** |  |  |  |
| Cigarettes, *number/day* | 0.321 | 0.384 | 0.610 |
| Global test | 0.339 | 0.620 | 0.324 |

**Supplementary Table S4.** Variance inflation factors (VIF) computed from the survival models (Applied to models of Table 3 and Table 5).

| Table 3 | **CV fatal and non-fatal events** | **All-cause death** | **ESKD** |
| --- | --- | --- | --- |
|  | **VIF** | **VIF** | **VIF** |
| Age | 2.13 | 2.14 | 2.13 |
| Gender | 3.41 | 3.42 | 3.42 |
| eGFR | 3.55 | 3.55 | 3.54 |
| Proteinuria | 1.34 | 1.33 | 1.34 |
| Current smokers | 1.24 | 1.24 | 1.23 |
| Former smokers | 1.74 | 1.74 | 1.73 |
| Previous CV disease | 1.58 | 1.58 | 1.57 |
| LDL-Cholesterol | 3.50 | 3.51 | 3.50 |
| Systolic blood pressure | 4.76 | 4.75 | 4.75 |
| Diabetes | 1.41 | 1.42 | 1.42 |
| **Table 5** |  |  |  |
|  | **VIF** | **VIF** | **VIF** |
| Cigarettes (Current smokers) | 2.74 | 2.73 | 2.73 |
| Cigarettes (Former smokers) | 2.39 | 2.39 | 2.39 |
